# Inhibition of mTORC2/Akt signaling to enhance the therapeutic potential of CD8 T cells

**DOI:** 10.1186/2051-1426-3-S2-P330

**Published:** 2015-11-04

**Authors:** Lianjun Zhang, Benjamin Tschumi, Susanne Oberle, Markus Ruegg, Michael Hall, Dietmar Zehn, Jean-Pierre Mach, Alena Donda, Pedro Romero

**Affiliations:** 1 Ludwig Cancer Research of University of Lausanne, Switzerland, Epalinges, Switzerland; 2 Swiss Vaccine Research Institute, Lausanne, Switzerland, Lausanne, Switzerland; 3 Biozentrum, University of Basel, Switzerland, Basel, Switzerland; 4 Department of Biochemistry, University of Lausanne, Switzerland, Epalinges, Switzerland

## 

CD8 T cells mediate protective immune responses against infections and cancer. Upon infection, antigen-specific naïve CD8 T cells are activated and differentiate into short-lived effector (SLEC) and memory precursor cells (MPEC). The T cell intrinsic signaling pathways underlying this differentiation remain largely unresolved. Here we show that Rictor, the core component of mammalian target of rapamycin complex 2 (mTORC2), regulates SLEC and MPEC commitment. Rictor deficient T cells form enhanced memory without dampening effector function, have increased IL-2 secretion capacity and mediate more potent recall responses. Mechanistically, enhanced memory formation in the absence of functional mTORC2 was associated with transcriptional and metabolic reprogramming by Eomes and Tcf-1 upregulation, repression of T-bet and nuclear stabilization of Foxo1. Elimination of Foxo1 reversed the increased MPECs differentiation and IL-2 production in Rictor KO mice. Effective T cell therapy against cancer depends highly on the generation of long-term persistent memory CD8 T cells. Our preliminary data show that Rictor deficient CD8 T cells show superior tumor protection effects in mouse melanoma model. Together, our study identifies mTORC2 as a central regulator of CD8 T cell differentiation and inhibition of mTORC2 or Akt might represent an effective strategy for both adoptive cell transfer and vaccine-based cancer therapies.

